# A hypothesis and evidence map for neuroimmune regulation of DOCK-family cytoskeletal programs in solid tumors

**DOI:** 10.3389/fimmu.2026.1927701

**Published:** 2026-08-25

**Authors:** Lei Zhou, Yuning Ren, Lina Han, Xiaodan Wang, Xiaoling Zhang

**Affiliations:** 1 Cancer Center, First Hospital of Jilin University, Changchun, China; 2 Key Laboratory of Organ Regeneration and Transplantation of Ministry of Education, First Hospital of Jilin University, Changchun, China; 3 National-Local Joint Engineering Laboratory of Animal Models for Human Disease, First Hospital of Jilin University, Changchun, China; 4 Department of Respiratory Medicine, Affiliated Hospital of Changchun University of Chinese Medicine, Changchun, China

**Keywords:** DOCK family GEFs, immune cytoskeletal paralysis, immune evasion, neuro-immune crosstalk, tumor microenvironment

## Abstract

The regulatory role of the nervous system in malignant tumor pathogenesis has emerged as an active and rapidly evolving field in tumor immunology. Within the tumor microenvironment (TME), innervation by the peripheral nervous system has been implicated not only in tumor initiation and progression but also in the establishment of an immunosuppressive network, partly through the reactivation of nerve-dependent developmental and regenerative programs. Although neural signals have been reported to promote immune cell exhaustion, the mechanisms by which they may directly influence the physical properties and biomechanical behavior of immune cells remain largely unexplored. In this review, we propose a testable hypothesis that neurotransmitters within the TME may engage receptors on immune cells and modulate the activity of the dedicator of cytokinesis (DOCK) family of atypical guanine nucleotide exchange factors (GEFs). This putative signaling axis could, in turn, disrupt DOCK-mediated actin cytoskeletal dynamics downstream of Rac/Cdc42, potentially contributing to cytoskeletal dysfunction, including impaired dendritic cell and macrophage chemotaxis through dense tumor stroma, and defective cytotoxic T-cell immune synapse formation. We synthesize current evidence supporting this framework, highlight critical mechanistic gaps, and outline an evidence map to guide future investigation. We also discuss the translational implications of targeting the proposed neural receptor-DOCK-cytoskeleton axis, while emphasizing that such applications remain speculative and require substantial experimental validation before any clinical translation can be justified.

## 1 Introduction

The tumor microenvironment (TME) has become a major focus of cancer research. It is composed of a complex network of stromal cells. The composition of stromal cells includes fibroblasts, immune cells, endothelial cells, pericytes, and mesenchymal stem cells (MSCs). The TME also contains non-cellular components, including the extracellular matrix (ECM), cytokines, and nutrients (1–4). Tumor progression and metastasis are regulated by multiple factors, including both tumor cell-intrinsic mutations and signals from the TME. The TME serves as a critical network that supports tumor survival, with its components regulating tumor initiation, progression, immune evasion, and therapy resistance (5). In this context, recent studies have confirmed that tumors are highly innervated tissues. The concept of solid tumor innervation has emerged as a rapidly evolving field in cancer research (6, 7). Most solid tumors arise outside the central nervous system (CNS) and receive neural innervation from the peripheral nervous system, especially the autonomic nervous system, which has been shown to regulate cancer progression across multiple tumor types (8). Evidence for the peripheral nervous system (PNS) innervation of solid tumors is accumulating. The mechanism of neural involvement is thought to involve perineural infiltration and axonal outgrowth, whereby peripheral nerve fibers, including sympathetic and sensory nerves, infiltrate tumor tissues. This phenomenon, termed tumor neoneurogenesis, is closely related to cancer progression. Numerous studies have confirmed that nerves are key promoters of signaling pathways involved in tumor growth and metastasis. Nerve fibers infiltrating the tumor not only promote tumor growth but also release abundant neurotransmitters (e.g., norepinephrine (NE) and CGRP), establishing a neural stress network at the tumor site and providing routes for cancer cell dissemination. Nerves, neurons, neurites, and neuroglia contribute functionally to tumor development. Neurotrophic factors (NTFs) and neurotransmitters act directly on receptors in tumor tissue to promote cell proliferation, while tolerogenic immune responses further facilitate tumor progression. The mechanism of neurogenesis involves tumor cells themselves releasing NTFs, which stimulate and induce the outgrowth of neural processes into the tumor, jointly participating in the TME. In a feedback response, the growing nerves also release neurotransmitters that subsequently trigger the migratory activity of tumor cells, resulting in tumor invasion and cancer metastasis (9). Research has shown that nerves not only have immune regulatory functions, but more importantly, they can regulate various signaling pathways of tumor cells and other cellular elements in the TME (10, 11). Thus, neurons and nerve fibers have become the core components of the TME. Therefore, in-depth research on the interactions between nerves, tumor cells, immune system, and other components of the TME will help design more precise targeted therapy strategies and gain a deeper understanding of the toxicity and resistance mechanisms associated with treatment.

Tumor-infiltrating nerves play a critical role in tumor progression by remodeling the TME and establishing bidirectional feedback with tumor cells. Therefore, quantitatively assessing the neural density within tumors is crucial for understanding their biological implications. Neuronal density, as a quantitative measure of neural distribution in tissues, is primarily increased in tumor stroma through axogenesis, neurogenesis, and neurotrophic effects (12). High neural density has been established as an adverse prognostic factor for many malignant tumors, including head and neck cancer (13), prostate cancer (14, 15), breast cancer (16) and colorectal cancer (17). In colorectal cancer, the intestinal mucosa exhibits extensive neural innervation, and the neural components in the TME play an important role in cancer progression. An increase in nerve density is significantly associated with poor prognosis in colorectal cancer patients, and denervation intervention can effectively inhibit tumor growth (18). Mechanistically, extensive tumor neurogenesis has been identified as an independent predictor of reduced overall survival in colorectal cancer (17). This oncogenic effect has also been validated in oral squamous cell carcinoma: high nerve density not only enhances tumor growth, but is also closely associated with poor survival and influences treatment choices in tongue cancer patients (19).

**Figure 1:**
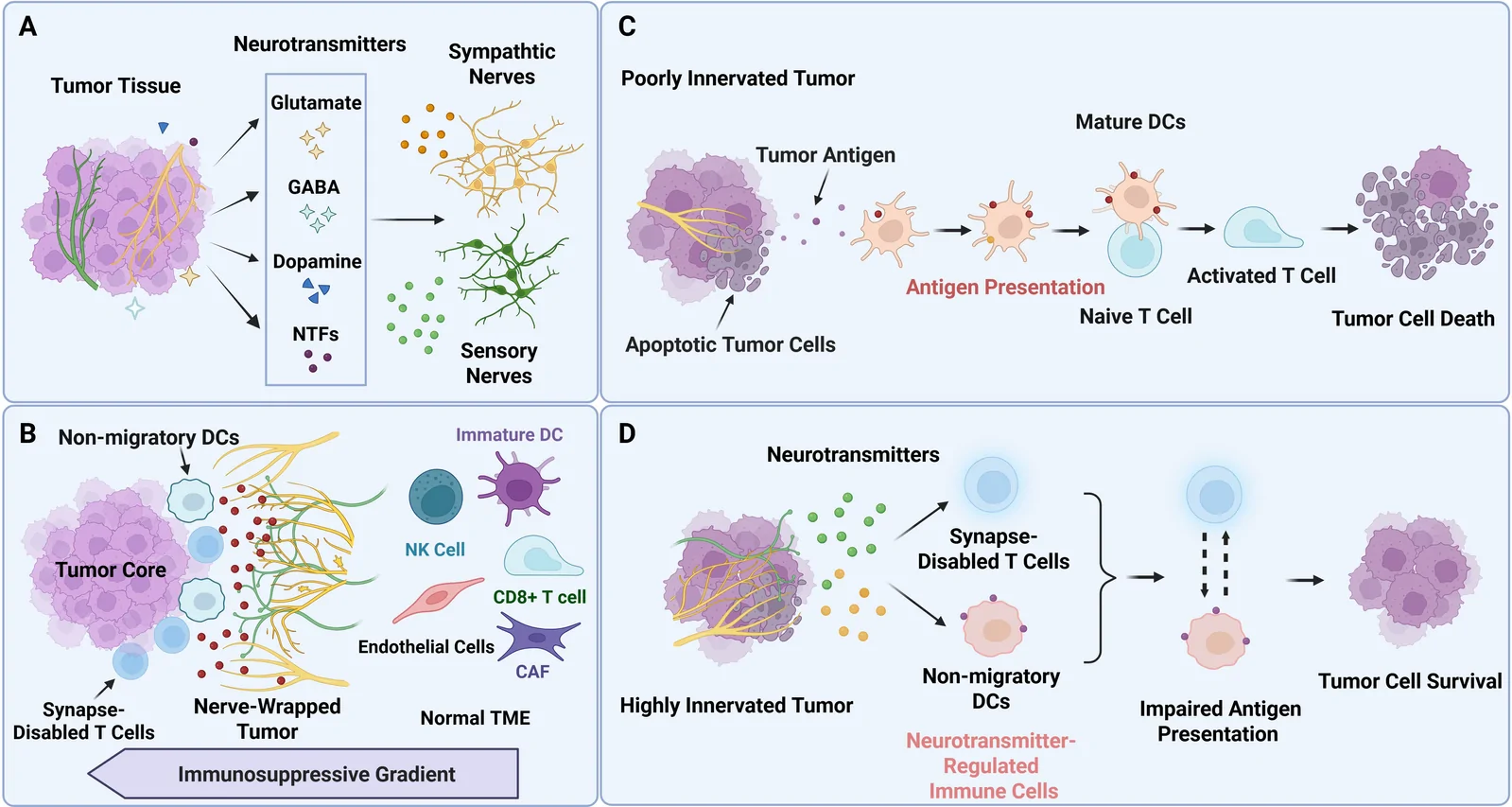
Tumor-nerve interaction mediates immune cell infiltration defect and dysfunction in the TME. **(A)** Tumor-released neurotransmitters induce innervation. Tumor cells actively secrete neuroactive factors including GABA, glutamate, dopamine, and neurotrophic factors (NTFs). Meanwhile, sympathetic and sensory nerves continuously release abundant neurotransmitters. Engagement of these mediators with their cognate receptors within the TME enables a close bidirectional crosstalk between the tumor and nerves, which in turn recruits sympathetic and sensory nerve fibers into the tumor stroma to form a dense neural network. **(B)** In tumor tissues with high innervation, the crosstalk between the tumor and nerves results in a gradient of neurotransmitters released by nerves, with concentrations increasing progressively toward the tumor. This gradient establishes a physical obstruction at the interface between the neural network and the tumor tissue. At this site, the migratory capacity of DCs is markedly impaired, and the formation of ISs between T cells and APCs or target cells is disrupted. Collectively, these events contribute to the establishment of a potent immunosuppressive TME. **(C)** In tumors with low innervation, DCs efficiently present tumor antigens, and activated T cells effectively clear tumor cells. **(D)** In tumors with high innervation, immune cells, including T cells and APCs (especially DCs), may be rendered dysfunctional by neurotransmitter signals, potentially disrupting antigen presentation and facilitating tumor immune evasion and survival.

High neural density within tumors is also a key factor leading to resistance to immune checkpoint blockade (ICB) therapy, and its mechanism involves multiple immunosuppressive mechanisms driven by aberrant neural-immune interactions (20–22). On the one hand, tumor infiltrating nerves activate the β-adrenergic signaling pathway by releasing neurotransmitters such as adrenaline and NE, directly regulating the PI3K/AKT/mTOR and NF-κB pathways in cancer cells to enhance their survival ability (23). It also reshapes the TME, promotes the recruitment and differentiation of immunosuppressive cells such as regulatory T cells (Tregs) and myeloid derived suppressor cells (MDSCs) (24). On the other hand, perineural invasion, as a direct communication platform between tumor cells and nerves, further exacerbates the immunosuppressive state (25), significantly inhibiting the infiltration and function of effector T cells in the TME. In addition, high neural density can enhance the immune escape ability of cancer cells against natural killer (NK) cells and cytotoxic T cells by upregulating the expression of immune checkpoint molecules such as programmed death-ligand 1 (PD-L1) on the surface of cancer cells (24). The formation of a “cold” TME driven by nerves limits the efficacy of ICB therapy centered around activated effector T cells (21, 26, 27) (**Figure 1**).

Recent studies have elucidated the direct molecular mechanisms underlying this neural-driven CD8^+^ T cell dysfunction. Globig et al. showed that exhausted CD8^+^ T cells upregulate β1-adrenergic receptors (ADRB1) and physically cluster around sympathetic nerves; catecholamine signaling through ADRB1 directly suppresses CD8^+^ T cell cytokine production and proliferation, and blocking this pathway limits T cell exhaustion and synergizes with immune checkpoint blockade (28). Similarly, chronic β-adrenergic signaling has been shown to impair CD8^+^ T cell proliferation, IFN-γ production, and cytolytic function, thereby reducing immunotherapy efficacy in preclinical lymphoma models (29). In addition to sympathetic signals, sensory CGRP^+^ neurons promote CD8^+^ T cell exhaustion through RAMP1 signaling in oral cancer, melanoma, and HNSCC, and higher RAMP1 expression on tumor-infiltrating lymphocytes correlates with poor patient survival (30, 31). These mechanistic insights provide a strong rationale for targeting neurotransmitter signaling pathways in cancer immunotherapy. Preclinical studies in mouse models have shown that β-blockade or inhibition of tumor neurogenesis can reverse immune suppression and enhance ICB efficacy (24). Retrospective epidemiological data have suggested potential benefits in ICB-treated patients, and early-phase clinical trials (e.g., NCT03384836) are currently evaluating the safety and efficacy of propranolol combined with pembrolizumab; however, definitive clinical benefit has not yet been established, and the combination remains investigational.

Traditional research on the regulation of immunity by the nervous system has been limited to the transcriptional and biochemical levels. For example, it has been revealed that sympathetic release of norepinephrine can directly induce T cell expression of programmed cell death protein 1 (PD-1), promote T cell exhaustion, or inhibit the secretion of inflammatory cytokines (32, 33). However, these mechanisms overlook a key physical prerequisite in anti-tumor immunity: immune cells must physically approach tumor cells and form mechanical contacts (i.e., immune synapses) in order to effectively exert their killing function. In the TME composed of dense matrix and mechanical stroma, the physical mobility of immune cells is as important as their cytotoxicity. Whether and how neural signals limit the recruitment and migration of immune cells from a physical and mechanistic perspective is currently a severely overlooked blind spot. Although existing evidence suggests that the sympathetic nervous system regulates immune cell differentiation and generation in the bone marrow and lymphoid organs (34–37), as well as directly blocking the recruitment of protective T cells to tumor tissue (38), this “recruitment deficiency” is often attributed to abnormal chemokine gradients. Beyond chemokine-mediated recruitment, DOCK8 has been shown to be critical for lymphocyte shape integrity during migration through confined spaces; DOCK8-deficient lymphocytes undergo a catastrophic cell death termed cytothripsis when navigating dense collagen-rich tissues, preventing the generation of long-lived tissue-resident memory CD8^+^ T cells (39). The emerging perspective of cancer neuroimmunology suggests that the nervous system may intervene in the behavior of immune cells through deeper physical mechanisms. For example, nerve fibers infiltrating tumors not only release neuroregulatory factors to shape the function of immune cells (40), but may also physically hinder the migration and infiltration of immune cells by altering the mechanical properties of the TME, such as promoting the contraction of cancer-related fibroblasts, reshaping the dense extracellular matrix, or directly regulating the mechanical perception and response ability of the immune cell cytoskeleton through neurotransmitters.

This physical immune barrier mediated by neural signals means that even if immune cells are not induced to a depleted state, they may lose their chance to fight due to their inability to travel to the tumor core in physical space. Therefore, elucidating how neural signals regulate the migration speed, matrix penetration, and stability of immune synapses of immune cells from the perspective of mechanobiology will be a key link in connecting the “neural-immune-tumor” three-dimensional network, and also provide a new physical target for overcoming immune therapy resistance.

Breaking through the dense physical barrier of solid tumors and performing contact killing, the immune synaptic function is a crucial factor that cannot be ignored (41). As the structural basis for direct killing of immune cells, immune synapses maintain immune homeostasis and prolong survival by enhancing cytokine secretion and improving tumor killing ability. Their function is highly dependent on the powerful mechanical force generation system within immune cells (42). In this system, the atypical GEF DOCK family (especially DOCK1, DOCK2, and DOCK8) plays a central role as a key regulator of the actin cytoskeleton. Through their GEF activity, DOCK proteins specifically activate Rho family small GTPases, thereby directly controlling the dynamic rearrangement of actin cytoskeleton (43–45). DOCK proteins are characterized by a catalytic DHR2 domain and a membrane-targeting DHR1 domain, with the DOCK-A and DOCK-B subfamilies constitutively associated with ELMO scaffold proteins that mediate auto-inhibition of DOCK GEF activity (46, 47). DOCK8 is a Cdc42-specific GEF that is crucial for the interstitial migration of DCs (48). Mechanistically, DOCK8 activation requires its DHR-1 domain to bind phosphatidylinositol 4,5-bisphosphate (PI (4,5) P_2_) at the plasma membrane, which is essential for its membrane recruitment and subsequent Cdc42 activation (49). In the absence of DOCK8, DCs lose their nuclear deformability and become trapped within the interstitial space which is dominated by dense fibrillar arrays of collagen bundles (50). Consequently, they are unable to effectively accumulate in the lymph node parenchyma to initiate T cell responses (48). Moreover, DOCK8 maintains lymphocyte shape integrity during migration through confined spaces; DOCK8-deficient lymphocytes undergo catastrophic cell death, termed cytothripsis, when navigating dense collagen-rich tissues, further impairing the generation of effective immune responses (39). Without DOCK2/8, cytotoxic T cells are unable to build the physical load-bearing wall composed of F-actin, leading to the mechanical breakdown of cytotoxic immune synapses. However, this powerful immune mechanical engine has a critical and potentially vulnerable “biochemical weakness” that is easily targeted by the TME. Early research suggests that the membrane localization and catalytic activity of DOCK and its adaptor protein ELMO1 (51) are highly dependent on their unphosphorylated conformational state. Specifically, the DOCK5-ELMO1 complex undergoes a conformational change on the plasma membrane driven by acidic lipids, adopting an open conformation (52). Mechanistically, this conformational change is not dependent on the phosphorylation state of ELMO1, but is primarily mediated by lipid interactions and hinge region rotation (52). Moreover, this conformational transition from closed to extended/open is critical for regulating the GEF activity of the DOCK5-ELMO1 complex at the plasma membrane. The activation of its catalytic capacity mainly relies on spatial rearrangement following membrane binding, rather than upstream phosphorylation events (52).

By contrast, structural studies on DOCK2-ELMO1 have indicated that phosphorylation can destabilize the auto-inhibited state and promote an active GEF conformation (47), suggesting that the regulatory mechanisms may be isoform-specific. Given that sympathetic neurotransmitters (e.g., norepinephrine) and neuropeptides signal through receptors that activate the cAMP/PKA pathway, it has been hypothesized that PKA might directly phosphorylate key sites such as ELMO1 or DOCK components. This hypothetical phosphorylation could, in principle, modulate the membrane localization and conformational state of DOCK complexes, potentially affecting the transmission of mechanical signals from membrane receptors to the cytoskeleton. However, direct evidence for this mechanism is currently lacking. In fact, the structural findings on DOCK2-ELMO1 described above point toward phosphorylation promoting an active conformation, which stands in contrast to the inhibitory model proposed here. The specific effect of PKA-mediated phosphorylation on DOCK family members may therefore be isoform- and context-dependent, and requires direct experimental validation. These observations raise the possibility that neural signals might suppress immune responses by modulating immune cell migration and synapse formation through DOCK/ELMO phosphorylation, but this hypothesis requires direct experimental validation and should not be interpreted as an established mechanism.

In this review, we propose a testable hypothesis that in highly innervated TME, neural signals may activate kinase cascades (e.g., PKA) in immune cells, which in turn could converge on DOCK-family GEFs to modulate cytoskeletal dynamics and impair immune cell infiltration and cytotoxic function.

## 2 Neural remodeling may drive the physical immune barrier in solid tumors beyond phenotypic exhaustion

### 2.1 The bottleneck of tumor immunotherapy: from phenotypic exhaustion to physical exclusion

Immune checkpoint blockade (ICB) therapy has been a milestone in modern cancer treatment. However, clinical responses remain limited: in most highly malignant solid tumors (such as liver cancer (53), pancreatic ductal adenocarcinoma (54), breast cancer (55, 56), colorectal cancer (57) and glioblastoma (58)), the response rate of ICB is still extremely limited due to challenges related to immunosuppressive microenvironment and cytotoxic T lymphocyte infiltration (53). For decades, efforts to overcome immune resistance have largely centered on phenotypic exhaustion at the biochemical and transcriptional levels, characterized by upregulation of inhibitory receptors (e.g., PD-1 (59), TIM-3 (60), LAG-3 (61), TIGIT (62)) and the loss of effector cytokine production (e.g., IFN-γ and TNF-α) (63–65). This approach attributes immune resistance to the fixed mindset of immune cells in receiving microenvironmental signals and their own transcriptional output. With the application of high-throughput imaging techniques such as spatial multiplex immunohistochemistry (IHC), researchers are able to simultaneously label multiple immune cells in the *in situ* TME. This allows us to not only quantify the abundance of immune cells, but also accurately analyze their spatial localization (such as tumor core, infiltrating edge, or stromal region) and the adjacent relationships between tumor and immune cells (66, 67). As a result, a more fundamental and undeniable mechanism of drug resistance gradually emerged, namely physical exclusion between spatial and mechanical dimensions. These findings have revealed an additional dimension of immune evasion: physical exclusion, providing a structural basis for understanding “cold” tumors (characterized by the absence or exclusion of tumor-infiltrating T cells) versus ‘hot’ tumors (characterized by active T cell infiltration). The classification of tumors as “cold” or “hot” is primarily determined by the extent of CD8^+^ T cell infiltration within the TME, rather than by the presence of other immunosuppressive cell types such as MDSCs or TAMs (68, 69), which may be enriched together with dysfunctional T cells in the immunosuppressive microenvironment. “Cold” tumors are characterized by limited T cell infiltration and are generally refractory to immune checkpoint blockade, whereas “hot” tumors exhibit robust T cell infiltration and are more likely to respond to immunotherapy (27). This distinction is clinically relevant, as the efficacy of ICB therapy is intrinsically linked to the presence of functional tumor-infiltrating T cells.

At the same time, clinical pathological observations have found that many “cold” tumors that are completely unresponsive to immunotherapy do not fail to induce systemic anti-tumor immune responses. Instead, in the tumor area, a large number of effector immune cells are blocked outside the tumor parenchyma, densely confined to the dense fibrous matrix around the tumor, forming a typical immune-excluded phenotype (70–73). This phenomenon highlights an unavoidable biophysical issue faced by immune cells, which is that if effector cells are unable to penetrate the matrix and come into contact with malignant cells at a physical distance, any intervention aimed at reversing their exhaustion state at the biological and chemical levels (such as simply using PD-1 antibodies) will become powerless and even be futile. In the past, a large amount of research tended to simply and conventionally attribute this physical isolation to the passive mechanical barrier formed by excessive proliferation of fibroblasts and collagen cross-linking (74–76). However, in recent years, in-depth analysis of the solid TME has revealed that this isolation is not only a passive structural barrier, but also a pathological state actively maintained by a highly active and highly regulated non-immune component in the TME, namely the peripheral neural network (77, 78). Emerging evidence suggests that this neural-driven barrier actively contributes to the formation of an immunosuppressive “cold” tumor microenvironment by limiting T cell infiltration (11, 26, 40, 79). The mechanisms by which neural signals drive this process will be discussed in detail in subsequent sections.

Solid tumors are not isolated cell populations, but highly organized new organs. They actively secrete signals such as neurotrophic factors and axonal directing molecules, inducing sympathetic, parasympathetic, and sensory nerve fibers to infiltrate and grow in a network-like manner toward the interior and edges of the TME, known as tumor neurogenesis (13). These abnormally dense nerve plexuses construct a high-intensity nerve stress barrier around the tumor periphery, continuously releasing high concentrations of peripheral neurotransmitters and neuropeptides (80, 81). These neurogenic signals not only indirectly exacerbate fibrosis of the matrix, but more importantly, they can directly engage homologous receptors on the surface of infiltrating immune cells (82–84), potentially modulating their mechanical properties and impairing immune cell migration and tissue penetration through cytoskeletal remodeling (85, 86). To overcome immune resistance in solid tumors, future strategies may need to address not only biochemical and phenotypic mechanisms but also the physical barriers imposed by neural-driven stromal remodeling.

### 2.2 Sympathetic and sensory nerves drive pro-tumorigenic networks at the neuro-immune interface in the TME

Previous studies have established that peripheral neurons are a key component of the TME, playing important regulatory roles in tumor occurrence, growth, metastasis, cancer-related pain, epithelial-mesenchymal transition (EMT), apoptosis resistance, and immune response (8, 87–89). Among them, the role of the autonomic nervous system in cancer has been fully elucidated, and recent studies have further revealed that sensory nerves are also involved. They negatively regulate the immune function in the TME by limiting the activation of protective anti-tumor immune responses, offering new therapeutic targets in melanoma (90). In pancreatic cancer, there is a mutual signal circuit between the pancreas and the nervous system including the central nervous system, which supports carcinogenic K-Ras induced tumor related inflammation. Pancreatic sensory neurons, as a key stromal cell population, not only promote the occurrence and progression of pancreatic ductal adenocarcinoma, but also may offer targets for early interventions in high-risk populations (91). With the development of neuroimmunology, we gradually realize that nerve fibers in the TME are not passive bystanders but form a complex communication network with infiltrating immune cells, namely the neural immune interface within the tumor. In this interface, sympathetic and sensory nerves directly modulate the host’s anti-tumor immune response by releasing specific neurotransmitters and neuropeptides, thereby inducing and maintaining a potent immunosuppressive microenvironment.

#### 2.2.1 Sympathetic nerve-driven immunosuppressive barriers

Under stress or tumor induced sympathetic nerve remodeling, the sustained release of NE and epinephrine (EPI) from sympathetic nerve endings into the TME drives the persistent activation of the β-adrenergic signaling axis (92–94). These catecholamines exert comprehensive inhibitory remodeling on innate and adaptive immune cells through β-adrenergic receptors (mainly β_2_-AR) on the surface of immune cells (95). Based on this characteristic, neurotransmitters released by nerve endings can effectively inhibit the anti-tumor function of immune cells. Specifically, on the one hand, when NE binds to the β_2_-AR on lymphocytes, it activates the cAMP/PKA signaling pathway within the cells, which not only directly inhibits T and NK cells proliferation and glycolysis metabolism, but also reduces the production of effector molecules such as IFN-γ and granzyme B (96–98). In addition, long-term sympathetic nervous signals stimulation can accelerate the evolution of CD8^+^ T cells toward exhaustion (28, 99). As the core force of anti-tumor immunity, the dual decline of CD8^+^ T cells in both quantity and function ultimately leads to a significant weakening of immune capabilities in the TME. On the other hand, sympathetic nervous signals also act as a brake pedal within the innate immune system, polarizing myeloid cells in a tumor-promoting direction. In particular, these signals induce tumor-associated macrophages (TAMs) to shift from the anti-tumor M1 phenotype to the pro-tumor M2 phenotype, accompanied by increased secretion of immunosuppressive cytokines such as IL-10 and TGF-β (100). Meanwhile, sympathetic activation significantly promotes the recruitment and expansion of MDSCs at the tumor site (101, 102).

#### 2.2.2 Sensory nerve-mediated immune evasion

Pathological alterations in neuropeptide signaling manifest as an imbalance between autonomic and sensory nerve functions (103). Specifically, the sympathetic nervous system, as a conduit for systemic stress responses, transmits stress signals to the tumor microenvironment (104–106). Meanwhile, sensory nerves establishing signaling connections act as local receptors and effectors of tumor, directly sensing and regulating homeostasis within the microenvironment (107, 108). Dysregulation of these signals is a critical driver of tumor progression. The neuropeptides released by sensory nerve endings play a crucial molecular bridging role in neuro-immune crosstalk. Calcitonin gene-related peptide (CGRP) can inhibit antigen presentation, and its mechanism is that the CGRP released by sensory nerves can bind to the receptor activity-modifying protein 1 (RAMP1) on the surface of DCs, langerhans cells (LCs) and macrophages (109–111), thereby effectively inhibiting the maturation and antigen presentation ability of DCs. This means that in areas where sensory nerves are active, tumor antigens cannot be effectively recognized and presented, thereby preventing the initiation of anti-tumor immune responses. CGRP can also reduce Th1 CD4^+^ T cells and activated CD8^+^ T cells in the TME by directly acting on the adaptive immune system (31, 112). Another important sensory neuropeptide, Substance P (SP) is a proinflammatory molecule that functions by interacting with its receptor, neurokinin-1 receptor (NK-1R) (113, 114). It can act on various immune cells to upregulate the proportion of Tregs, and enhance local immune tolerance in conjunction with other factors, thereby participating in shaping the immunosuppressive microenvironment. Sensory nerves can directly induce T cell dysfunction. Recent studies have shown that sensory nerves in TME can even form synaptic-like physical contact with CD8^+^ T cells, inducing T cell expression of inhibitory receptors (such as PD-1 and TIM-3) through local release of high concentrations of neuropeptides, accelerating immune escape (112).

#### 2.2.3 Neuro-immune synapse-like structures and their accompanying microenvironmental metabolic crosstalk

In the microenvironment of solid tumors, the interaction between nerve fibers and immune cells is not solely dependent on the distant paracrine secretion of soluble factors, but is established on highly specific spatial anatomical contacts. Previous studies have confirmed through confocal microscopy that there is close spatial contact between SP positive nerve fibers and SP positive thymocytes, indicating that the immune system and nervous system can communicate through a series of signaling molecules including SP (115). About 10% of DCs were found to be in contact with nerve endings in allergic airway inflammation, with up to 4% of T cells forming cell clusters with these DCs (116). High resolution spatial omics and electron microscopy imaging show that reshaped sympathetic and sensory nerve endings can have extremely close physical contact with TAMs (105, 117), DCs (118, 119), and even T cells (120). In the spatial structure of solid tumors, nerve fibers and immune cells often exhibit significant spatial colocalization, forming gaps that are only nanoscale neuro-immune synapse-like structures (7, 121). The neuro-immune synapse has bidirectionality, and cytokines secreted by immune cells can enhance sympathetic nervous activity by acting on the central nervous system, thereby promoting the mobilization, migration, and infiltration of immune cells in terminal organs (122). This dense neural immune interwoven network forms a physical immune barrier in the local area of solid tumors. The neuro-immune synapse-like structure not only limits the deep infiltration of effector T cells into the tumor core area in terms of spatial hindrance, but more importantly, it provides an excellent anatomical basis for the targeted and high concentration transmission of neurotransmitters. Accompanying this high-frequency physical contact is the local deep metabolic crosstalk in the microenvironment.

In the microscale space of neural immune synapses, the continuous input of neural signals profoundly reshapes the metabolic programming of immune cells. On the one hand, the high concentration of norepinephrine released by sympathetic nerve endings not only directly activates the β_2_-AR of target cells, but also exacerbates spatial hypoxia by regulating local microvascular tension. This dual deprivation of hypoxia and neural signals forces infiltrating T cells to undergo metabolic reprogramming, accelerating their metabolic exhaustion. On the other hand, neuropeptides (e.g., CGRP) released locally by sensory nerve endings can alter the lipid metabolism pathway of DCs, leading to abnormal accumulation of lipids inside the cells and directly weakening their antigen cross presentation ability. Therefore, the synaptic like structure at the physical level and the deterioration of the metabolic microenvironment at the chemical level are intertwined, jointly locking in the local immune tolerance state of solid tumors.

#### 2.2.4 New strategies and translational insights for clinical combination therapy targeting the neural immune interface

The central and peripheral nervous systems jointly regulate the pathophysiological processes of tumors (7, 123). In particular, the CNS regulates tumor growth and metastatic ability through its interaction with the immune system (124). It is noteworthy that the migration of CNS-derived neural progenitor cells from the central nervous system to peripheral tumors also indicates the active involvement of neural stem cells in tumor initiation and progression (125). The tight connection between immune cells and nerve endings that govern immune organs allows the immune system to also mobilize local neurons to influence immune responses (7). The physical barrier formed by the highly dense neural immune interface in solid tumors not only hinders the deep infiltration of effector T cells into the tumor, but also interferes with the continuous release of neurotransmitters and metabolism by nerve fibers, accelerating the phenotypic exhaustion of infiltrating T cells within the TME. This highly dominated immune tolerance state by the nervous system is considered one of the key driving factors for the development of primary or secondary resistance to immune checkpoint inhibitors such as PD-1/PD-L1 therapy in the current body. Therefore, breaking down this strong barrier of neural immune construction has become a highly promising new direction for clinical translation.

In terms of specific joint intervention strategies, intervention strategies targeting the sympathetic nervous axis has shown clinical potential for the first time. In immune cells, sympathetic driven metabolic changes induce the formation of immunosuppressive phenotypes, especially in T cells and macrophages (126). Studies have confirmed that blocking sympathetic nervous system (SNS) signaling is mainly achieved through β-AR, which enhance immune cell function by increasing CD8^+^ T cell activation and cytokine production, while reducing the population of immunosuppressive cells (127). β blocker propranolol treatment can reduce the frequency and immunosuppressive function of TAMs, and enhance anti-tumor immunity macrophages in malignant pleural effusion exhibit an immunosuppressive phenotype by activating the norepinephrine metabolic pathway (128). The combination of nonspecific β blocker propranolol and ICIs have recently been used to treat metastatic angiosarcoma. Mechanistically, propranolol treatment leads to upregulation of PD-L1 on TAMs and changes in their chemokine expression profiles. The combination administration of propranolol significantly improves the efficacy of anti-CTLA4 therapy (129). This indicates that this combination therapy can also effectively block the cAMP/PKA inhibitory signaling induced by catecholamines, not only reversing the tumor promoting polarization of macrophages, but also breaking down the immunosuppressive barrier from both physical structure and spatial metabolism levels by improving local microvascular tension and hypoxia status.

Meanwhile, precise interventions targeting sensory nerves have also made breakthroughs in preclinical research on tumor treatment. The communication between the nervous system and the immune system involves the release of neuropeptides, including CGRP, from sensory nerves during inflammation (130). Conventional type 1 dendritic cells (cDC1s) show high expression of CGRP receptors (131). CGRP inhibits the expression of pro-inflammatory mediators, including TNF-α and CCL4, in DCs via the cAMP/PKA signaling pathway (130). By using CGRP receptor antagonists or local denervation techniques, the restriction of neuropeptides on DC antigen presentation function can be significantly relieved, thereby rescuing the anti-tumor immune response on the brink of exhaustion at the source.

In summary, the combination of targeted neural level regulatory interventions and classical immunotherapy essentially eliminates the protective neural network of tumors through pharmacological means, relieves the blocking effect of neural signals on the immune system, and makes it easier for T cells to infiltrate deep into the tumor. This strategy is expected to successfully reverse cold tumors that originally lacked immune response into hot tumors, providing a new solution to overcome the bottleneck of solid tumor immunotherapy.

## 3 The central role of DOCK protein family in regulating the structure of immune cells

The DOCK family consists of 11 members, all of which contain two domains: DOCK homologous region (DHR) -1 and DHR-2 (132). Unlike other GEFs, DOCK family proteins do not contain the common Dbl homologous domain, but they mediate the GTP/GDP exchange reaction through the DOCK DHR-2 domain (133). The DHR2 domain is a catalytic structure consisting of approximately 450 residues located in its C-terminal region (134, 135). The DHR1 domain of DOCK protein adopts a C2-like structure and interacts with PI (3,4,5) P3, mediating signal transduction and membrane localization (136, 137). As a member of the GEF family, it can regulate intracellular actin dynamics by activating Rho GTPases CDC42 and RAC, thereby participating in Rho GTPase dependent cellular processes (46, 47). The 11 members of the DOCK family can be divided into four subfamilies based on their sequence and substrate specificity: DOCK-A (DOCK1, 2, and 5), DOCK-B (DOCK3 and 4), DOCK-C (DOCK6, 7, and 8), and DOCK-D (DOCK9, 10, and 11). DOCK family proteins play a crucial role in immune regulation, including immune cell development, survival, migration, activation, tolerance (132, 133) (**Table 1**).

**Table 1:** Summary of immunomodulatory characteristics of DOCK family members.

DOCK subfamily/member	Target GTPase	Key immune cell types	Physiological cytoskeletal function	Neuro-stress link (hypothesis/proposed)	Proposed TME consequence	Evidence level for neuro-stress link	Key refs.
DOCK-A							
DOCK2 (Critical)	Rac1, Rac2	T cells, B cells, DCs, Neutrophils	Regulates actin cytoskeleton dynamics to drive immune cell migration and IS formation; controls megakaryocyte differentiation, proplatelet formation, and platelet cytoskeletal assembly, thereby influencing hemostasis.	β-AR/cAMP/PKA signaling has been hypothesized to modulate DOCK2-ELMO1 function, but direct evidence is lacking. Structural evidence suggests phosphorylation may favor an active conformation.	Impaired infiltration; dysfunctional immunological synapses.	Inferred/Absent	(221–223)
DOCK1, DOCK5	Rac1	Macrophages, Neutrophils	Mediates phagocytosis and cell motility.	PKA-dependent phosphorylation of DOCK1 at S1250 has been shown to promote Rac1 activation in glioma cells (not in immune cells). For DOCK5, direct evidence is lacking.	Impaired phagocytosis of tumor debris by macrophages; defective innate immune surveillance.	Demonstrated in tumor cells (DOCK1); Absent (DOCK5)	(147, 224, 225)
DOCK-C							
DOCK8 (Critical)	Cdc42, Rac1	DCs, T cells, NK cells	Essential for amoeboid migration of DCs through dense 3D matrices; maintains T/NK cell integrity and generates synaptic.	It has been proposed that neural stress signals might compromise DOCK8 function, but direct evidence for neurotransmitter-dependent DOCK8 phosphorylation, Cdc42 activation, or F-actin remodeling is lacking.	DCs trapped in dense tumor stroma, failing to reach lymph nodes for antigen presentation. Cytotoxic cells form contacts but fail to secrete lytic granules.	Inferred/Absent	(48, 163, 226)
DOCK6; DOCK7	Cdc42, Rac1	Primarily neuronal.	Regulates neurite outgrowth; potential roles in regulating specific immune cell morphology.	Potential crosstalk with axon guidance molecules expressed in TME; this remains speculative.	May contribute to aberrant neuro-immune cell interactions.	Inferred (non-immune cell evidence)	(227, 228)
DOCK-B							
DOCK3; DOCK4	Rac1, Rap1	Various leukocytes, Endothelial cells,	Regulates cell-cell adhesion junctions and vascular integrity.	Stress signaling may modulate Rap1 activity via DOCK4, but direct evidence for this in the TME is lacking.	Altered vascular permeability affecting immune cell extravasation.	Inferred (pharmacological inference)	(229, 230)
DOCK-D							
DOCK9; DOCK10; DOCK11	Cdc42, Rac1	B cells, Macrophages	Regulates B cell activation, macrophage fusion, and shape changes, and induces Rac1-dependent membrane ruffling.	Potential modulation of macrophage polarization states.	May contribute to the morphological shaping of pro-tumorigenic M2-like macrophage subsets.	Inferred/Absent	(171, 231, 232)

Evidence level definitions: ‘Demonstrated in tumor cells’ indicates direct experimental evidence from tumor cell lines; ‘Demonstrated in immune cells’ indicates direct experimental evidence from immune cell systems; ‘Inferred (non-immune cell evidence)’ indicates a plausible hypothesis based on evidence from non-immune cell types (e.g., neuronal cells); ‘Inferred (pharmacological inference)’ indicates a plausible hypothesis based on pharmacological or indirect evidence; ‘Absent’ indicates that no direct evidence currently exists for the proposed neuro-stress link in the specified context.

### 3.1 The role of DOCK-A subfamily in the structure and function of immune cells

The DOCK-A subfamily (DOCK1, DOCK2, and DOCK5) plays a central role in immune cell migration, phagocytosis, and immune synapse formation. Among these, DOCK2 is the most extensively characterized member in the immune system. Expressed predominantly in hematopoietic cells, DOCK2 coordinates lymphocyte trafficking and homing by mediating chemokine-induced Rac activation and actin polymerization (138–140). In addition, DOCK2 is indispensable for immune synapse formation in T cells, B cells, and NK cells through the Rac-actin cytoskeleton axis (141–146). DOCK1 and DOCK5, which are more ubiquitously expressed, regulate myeloid cell functions, particularly Fcγ receptor-mediated phagocytosis in macrophages and chemotaxis in neutrophils, through Rac-dependent cytoskeletal remodeling (147–152). The detailed expression patterns, specific functions, regulatory mechanisms, and immune consequences of each DOCK-A member are summarized in **Table 1**.

### 3.2 The role of DOCK-B subfamily in the structure and function of immune cells

The DOCK-B subfamily (DOCK3 and DOCK4) is also involved in cytoskeletal dynamics and immune cell function, although their roles in tumor immunology are less extensively studied compared to DOCK2 and DOCK8. DOCK3 has been implicated in tumor cell migration and metastasis across multiple cancer types, including colorectal cancer, small cell lung cancer, melanoma, and prostate cancer (153–158). DOCK4 serves as a prognostic biomarker in stomach adenocarcinoma and colon adenocarcinoma, and may play a role in regulating immune cell infiltration and tumor microenvironment remodeling (154, 159–161). See **Table 1** for a detailed summary of their expression, functions, and associated immune consequences.

### 3.3 DOCK-C subfamily in the structure and function of immune cells

The DOCK-C subfamily (DOCK6, DOCK7, and DOCK8) is dominated by DOCK8, which occupies a central position in immune cell structure, function, and disease. DOCK8 deficiency causes autosomal recessive hyper-IgE syndrome (AR-HIES), leading to combined immunodeficiency in humans, highlighting its essential role in immune homeostasis (162). Mechanistically, DOCK8 regulates immune synapse formation in T and B cells by linking Cdc42 activation to the WASP-Arp2/3 actin polymerization pathway (162, 163). DOCK8 is also essential for dendritic cell amoeboid migration through dense matrices (48), and for LFA-1-dependent T follicular helper cell localization in germinal centers (164). At the pathophysiological level, DOCK8 deficiency impairs T cell proliferation, cytokine production, and B cell differentiation, while also affecting metabolic reprogramming in CD4^+^ T cells via the Akt/mTOR/HIF-1α pathway (39, 165–168). Clinically, high DOCK8 expression correlates with favorable prognosis and increased immune infiltration in HPV-positive head and neck squamous cell carcinoma (169, 170). A comprehensive summary of DOCK6, DOCK7, and DOCK8 expression, functions, regulatory mechanisms, and immune consequences is provided in **Table 1**.

### 3.4 DOCK-D subfamily in the structure and function of immune cells

The DOCK-D subfamily (DOCK9, DOCK10, and DOCK11) has received relatively less attention in immune cell biology compared to DOCK2 and DOCK8. DOCK10, the best-characterized member of this subfamily, regulates actin cytoskeleton remodeling and induces membrane ruffling and filopodia formation (171). In B cells, DOCK10 expression is regulated by IL-4 signaling and is associated with B cell malignancies (171). In innate immune cells, DOCK10 deficiency impairs macrophage and microglia migration, and reduces TLR-induced CCL2 expression in astrocytes (172). These findings implicate DOCK10 in neuroinflammatory processes, suggesting its potential as a therapeutic target for neuroinflammatory diseases such as multiple sclerosis (172). Detailed information on DOCK9, DOCK10, and DOCK11, including expression patterns, functions, and immune consequences, is presented in **Table 1**.

Collectively, the cytoskeletal remodeling of immune cells in the innervated TME likely involves a multi-layered regulatory network, encompassing both DOCK-dependent pathways (via Rac/Cdc42 activation) and DOCK-independent mechanisms (such as stress hormone-induced modulation of ERM/actin-binding proteins). The DOCK family, particularly DOCK2 and DOCK8, plays a central role in Rac/Cdc42-mediated actin dynamics, as summarized in **Table 1**. However, DOCK-mediated pathways are not the sole route through which neurogenic and stress signals modulate the immune cell cytoskeleton. Flint et al. demonstrated that stress hormones, including cortisol, NE, and epinephrine (E), directly alter the actin cytoskeleton in T lymphocytes by modulating the expression and subcellular distribution of moesin and other actin-binding proteins (173). Exposure to these hormones induced a marked redistribution of moesin and actin toward the distal pole complex, accompanied by a loss of CD43, a negative regulator of T cell activation. This moesin/ERM-dependent pathway operates in parallel to the DOCK-Rac-actin axis and may represent an additional layer of regulation through which neural-derived signals impair T cell migration and function, particularly under chronic stress conditions in the TME.

It is also important to note that, while the DOCK-Rac/Cdc42-actin axis is well established in immune cell biology, the upstream neural inputs that may regulate this pathway in the TME remain largely hypothetical. To date, no direct evidence links specific neural receptors (e.g., α2-AR, CGRPR) to PKA-mediated DOCK phosphorylation in tumor-infiltrating immune cells. Most of the proposed mechanistic steps are inferred from indirect or pharmacological studies and require direct biochemical and structural confirmation. Thus, the neuroimmune regulation of DOCK-family cytoskeletal programs should currently be viewed as a testable hypothesis and evidence map, rather than an established mechanism a framework that will guide the discussion in the following sections.

## 4 Neural regulation of GEF-mediated signal transduction from receptors to the cytoskeleton

### 4.1 Alpha2-adrenergic receptors inhibit the cAMP/PKA pathway

**Figure 2:**
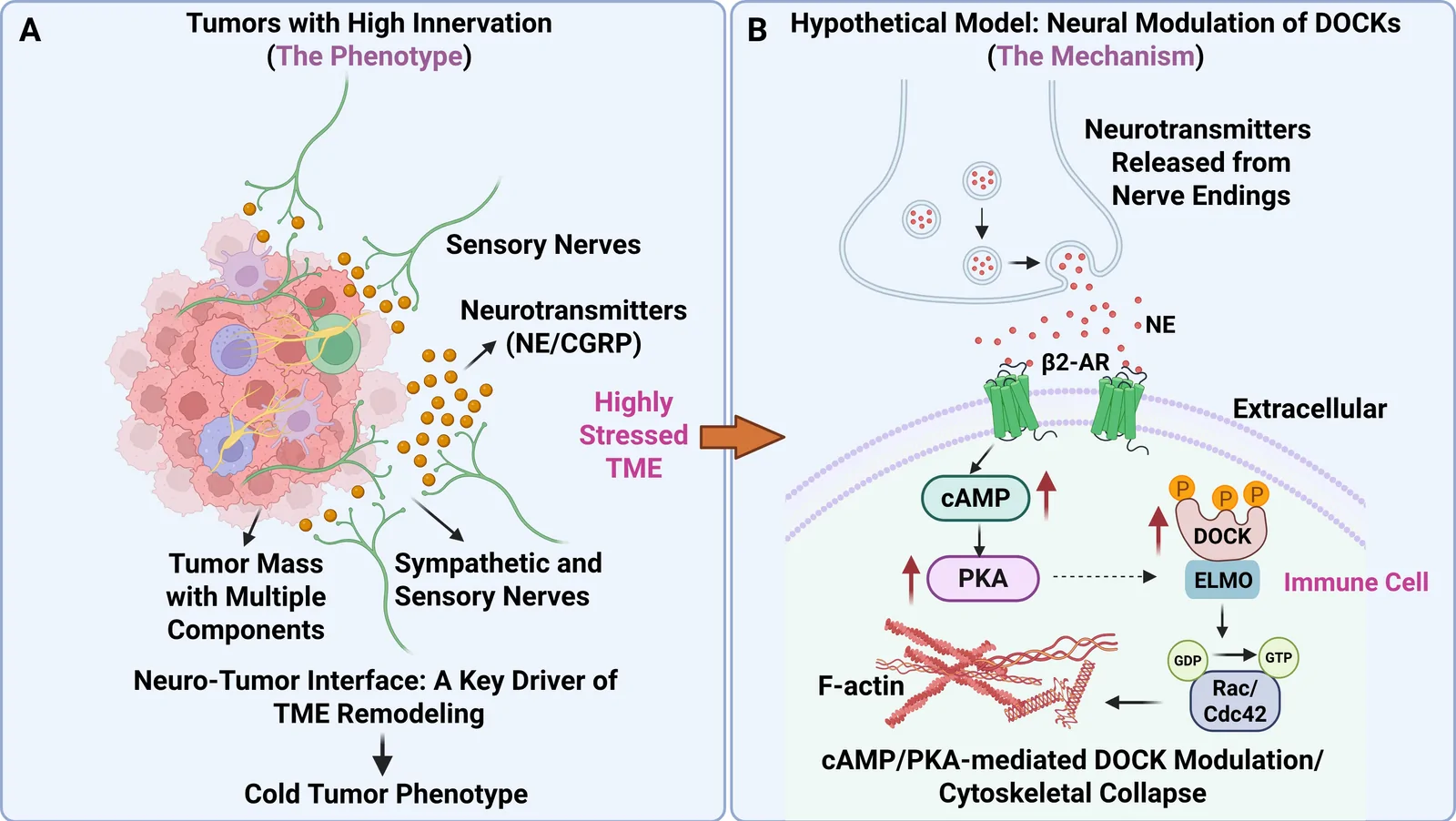
Tumor-nerve interaction plays a critical role of TME remodeling. **(A)** Under high innervation, sympathetic and sensory nerves release abundant neurotransmitters (including NE and CGRP) into the TME. This establishes a neuro-tumor interface that contributes to immune cell exclusion and a ‘cold’ tumor microenvironment, characterized by limited T cell infiltration **(B)** Proposed model for neuroimmune regulation of DOCK-family cytoskeletal programs in the TME. Solid lines indicate steps supported by experimental evidence; the dashed line from PKA to DOCK indicates a hypothesized step that currently lacks direct experimental confirmation in immune cells. For isoform-specific evidence, see **Table 2**. This model is presented as a testable hypothesis to guide future investigation.

The GPCR/cyclic adenosine monophosphate (cAMP)/protein kinase A (PKA) pathway is one of the most common and functionally diverse signaling pathways in eukaryotic cells (174). Epinephrine and norepinephrine can activate alpha2-AR to exert inhibitory cellular responses, some of which are due to the decrease in cAMP caused by the inhibition of adenylate cyclase (175). During the pathogenesis of chronic cholestatic liver diseases, alpha2-AR agonists can inhibit the prolactin-stimulated increase in cAMP levels and PKA activity, thereby counteracting the enhanced ductal secretion associated with cholangiocyte proliferation (176). In corneal epithelial and endothelial cells, unlike beta-adrenergic stimulation which activates adenylyl cyclase and subsequently PKA, alpha2A-adrenoceptor stimulation inhibits PKA activity by suppressing adenylyl cyclase (174). Norepinephrine induces melanophore apoptosis by attenuating the cAMP-PKA signaling pathway through activation of alpha2-AR (177). The multiple studies mentioned above have confirmed that activation of alpha2-AR can reduce intracellular cAMP levels and inhibit PKA activity by suppressing adenylyl cyclase (**Figure 2**). The pharmacological basis of alpha2-AR signaling is well established; however, direct evidence linking this pathway to DOCK family function or cytoskeletal regulation in immune cells is currently lacking.

### 4.2 PKA signaling and DOCK-family GEFs: a hypothesis for neuroimmune cytoskeletal regulation

Studies have confirmed that PKA phosphorylates DOCK1 at serine residue 1250, and that this phosphorylation promotes Rac1 activation and cell migration in glioma cells (178). Similarly, EGFRvIII has been shown to phosphorylate Dock1 via PKA, driving glioma progression, suggesting that the EGFRvIII-PKA-Dock180-Rac1 axis may represent a potential therapeutic target for glioma. However, it is important to note that these observations are derived primarily from tumor cell lines; whether similar PKA-mediated DOCK1 phosphorylation occurs in immune cells, and what its functional consequences might be, remains unknown. Thus, the available evidence does not justify a generalized conclusion that PKA phosphorylates all DOCK family members to regulate Rac/Cdc42.

In contrast to the direct evidence linking PKA to DOCK1, evidence for PKA-mediated phosphorylation of other DOCK isoforms remains limited. For DOCK2, structural studies indicate that phosphorylation can destabilize the auto-inhibited state and favor an active GEF conformation, but the relevant kinase has not been definitively identified in immune cells. For DOCK5, membrane-driven conformational changes are critical for GEF activity, but direct evidence for PKA-mediated DOCK5 phosphorylation is lacking. For DOCK8, PKCα-mediated phosphorylation has been reported to regulate T-cell migration, but no direct link to PKA has been established. It has been speculated that PKA may be involved in cytoskeletal regulation through spatial localization, as multiple A-kinase anchoring proteins (AKAPs) can associate PKA with actin cytoskeletal components (179). Elevated cAMP and PKA activation are essential for filopodia/lamellipodia formation (180), cell migration on laminin (181), microfilament assembly (182), and Rac and Cdc42 activation (183, 184). However, PKA does not directly phosphorylate Cdc42 (183), suggesting that upstream GEFs, potentially including certain DOCK family members, may mediate PKA effects on Cdc42. This possibility warrants investigation but remains speculative at present.

The adaptor protein engulfment and cell motility 1 (ELMO1)/the DOCK1 complex, which functions as a GEF for Rac1, participates in cell adhesion, migration, and phagocytosis. This complex can interact with the GPCR GPR124/ADGRA2 and regulate cytoskeletal dynamics during endothelial cell adhesion (185). Extracellular adenosine has been shown to enhance endothelial barrier function through an ELMO1/Rac1/PKA signaling mechanism (186). However, these findings do not establish a direct link between PKA and ELMO1/DOCK phosphorylation; rather, they suggest that PKA and ELMO1/Rac1 may function in parallel or intersecting pathways. The possibility that the ELMO/DOCK complex acts as a regulatory node for Rac1 within PKA-related signaling pathways remains speculative.

While Alpha2-AR has been shown to regulate cellular functions through the cAMP/PKA pathway in certain cell types (e.g., in retinal ganglion cells, a PKA inhibitor can block adrenergic regulation (187)), direct evidence linking Alpha2-AR to PKA-mediated DOCK phosphorylation or Rac/Cdc42 activation in immune cells is currently lacking. It is tempting to speculate that sympathetic signaling, through modulation of PKA activity, might influence DOCK-mediated Rac/Cdc42 activation. However, this hypothetical framework requires direct experimental validation, particularly in tumor-infiltrating immune cells, and should not be interpreted as an established pathway. Similarly, there is currently no direct evidence linking specific sensory neuropeptides (e.g., CGRP, SP, or NMB) to the activation of DOCK family proteins downstream of their GPCRs, nor has direct phosphorylation of DOCK family members by PKA in sensory neurons been reported. These represent important gaps for future investigation.

Together, these findings suggest a working hypothesis: neurotransmitters and neuropeptides released in the TME may engage GPCRs on immune cells, modulating PKA activity and thereby affecting the phosphorylation and GEF function of specific DOCK isoforms. This could alter Rac/Cdc42-dependent actin dynamics and influence immune cell migration, synapse formation, and effector function. This hypothesis remains to be tested, but it offers a framework for investigating how neural signals regulate immune cell behavior at the cytoskeletal level.

## 5 Dual failure of lymphocyte synapse and myeloid motility in the TME

### 5.1 The collapse of the immunological synapse

**Figure 3:**
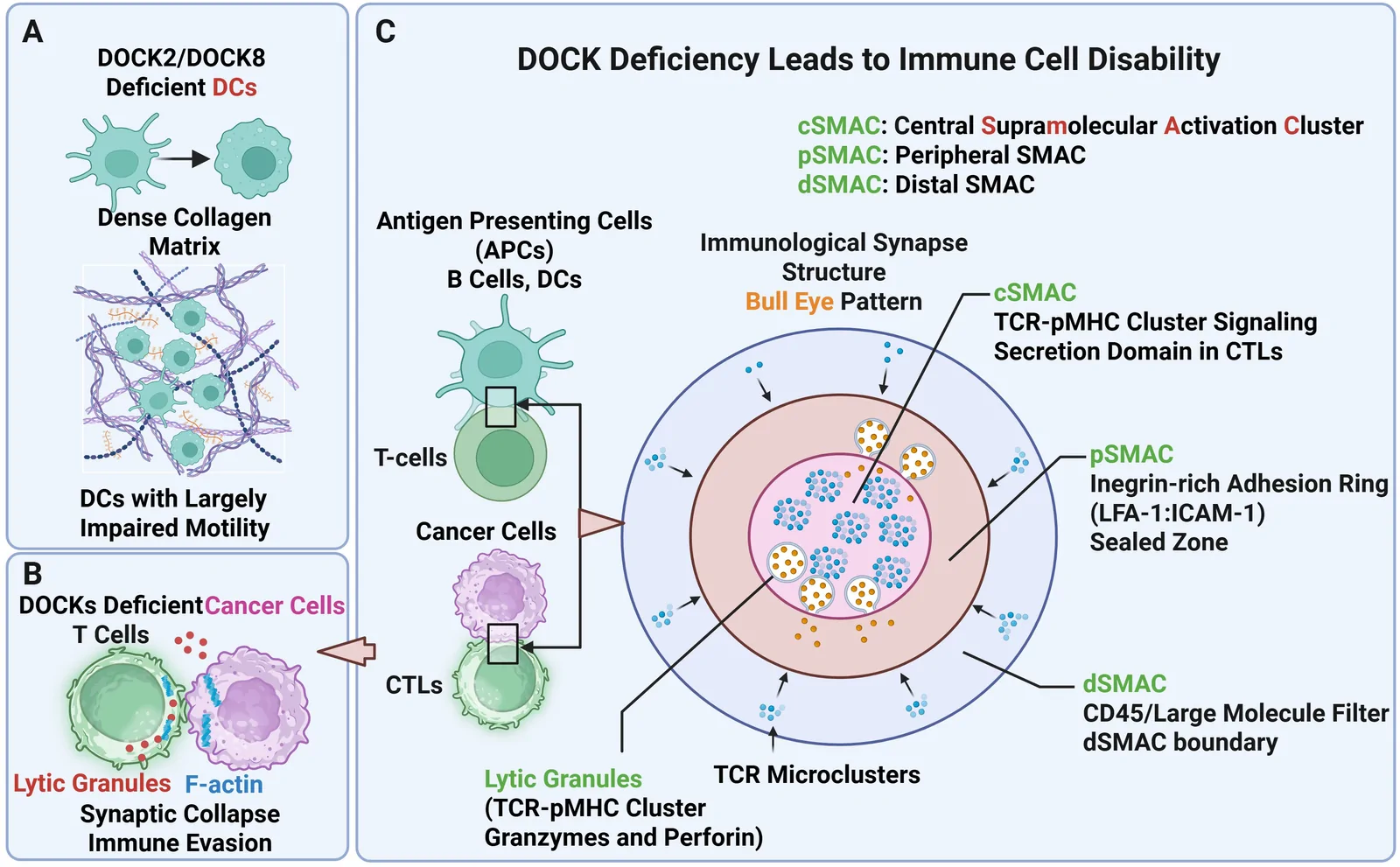
Deficiency of DOCK family members impairs DC migration and disrupts T cell ISs formation. **(A)** When DCs lack DOCK2/DOCK8, their migratory capacity within dense collagen matrices is significantly impaired. **(B)** DOCK-deficient T cells show impaired immunological synapse formation with tumor cells, as defective F-actin polymerization in T cells disrupts the cytoskeleton and compromises lytic granule release, ultimately leading to IS dysfunction and facilitating tumor immune evasion. **(C)** Anatomical diagram of IS structures between CTLs and tumor cells, and between T cells and APCs.

In the process of immunological synapse formation, when cytotoxic T cells come into contact with antigen-presenting cells (APCs) or tumor target cells, the assembly of actin and actomyosin networks in the contact region is significantly enhanced (190). This process is accompanied by extensive remodeling of the actin cytoskeleton and is highly dependent on sustained actin polymerization (treadmilling). As remodeling progresses, three characteristic regions form within the mature immunological synapse: the central supramolecular activation cluster (cSMAC) and the surrounding peripheral SMAC (pSMAC) and distal SMAC (dSMAC) (191, 192). The establishment of this structure is crucial for achieving intercellular communication and cytotoxic function. During this process, the polarization of the MTOC toward the synaptic contact site ensures the directional secretion of cytokines and cytolytic molecules (193). From a mechanistic perspective, molecules such as DOCK2 and DOCK8 are core regulators of synaptic actin dynamics. Studies have shown that mutations in DOCK2 and DOCK8 lead to defective immunological synapse formation in T cells and impair LFA-1 recruitment (194). Mechanistically, they influence the formation, stability, and function of the IS by regulating small GTPases such as Rac and Cdc42 (**Figure 3**).

Given that DOCK2 and DOCK8, prominent members of the DOCK family, play crucial roles in IS formation and function, we propose a hypothesis: neurotransmitters enriched in the TME may disrupt proper immunological synapse assembly by inhibiting the activity of DOCK2/8, thereby impairing the ability of T cells and even NK cells to effectively eliminate tumor cells upon contact, a function normally performed in healthy states. Currently, no literature directly confirms this complete hypothesis. However, we have mastered three independent research areas and have summarized compelling indirect evidence supporting them in different chapters of this paper: (1) the phenomenon of neurotransmitter enrichment in the TME [section 1.1, 1.2]; (2) the core function of DOCK2/8 in regulating immunological synapses in immune cells (particularly T cells and NK cells) [section 2.1, 2.3]; and (3) the established role of neurotransmitters in modulating immune function. Integrating these three lines of evidence establishes a solid theoretical foundation for our proposed novel regulatory mechanism.

The function of neurotransmitters as signaling molecules extends beyond traditional understanding; they are not only signal transmitters within the nervous system but also key regulators of systemic homeostasis. Neurotransmitters mediate signals through specific receptors on the surface of immune cells, coordinating immune responses via the crosstalk between the nervous and immune systems. In this way, they regulate the interactions among the nervous, immune, and metabolic systems, profoundly influencing the development of various pathological processes, including cancer, neurodegenerative diseases (NDDs), and inflammatory disorders (195).

During tumor progression, a variety of neurotransmitters play critical roles by reshaping the immune microenvironment, such as EPI and NE (80), serotonin (5-HT) (196), acetylcholine (ACh) (197), and CGRP (198), etc. Catecholamines (EPI/NE) are the most extensively and deeply studied immunomodulatory neurotransmitters. The vast majority of immune cells, including T cells, B cells, NK cells, macrophages, and dendritic cells, express β-ARs (199). EPI and NE exert immunosuppressive effects by binding to these receptors, thereby promoting tumor immune evasion. Mechanistic studies have shown that activation of β-AR signaling significantly inhibits the proliferation, IFN-γ production, and cytolytic killing capacity of antigen-specific CD8^+^ T cells (29). Furthermore, NE induces IL-6 secretion through a β2-AR-dependent mechanism, promoting M2 macrophage polarization and subsequently enhancing the migratory capacity of cancer cells (200). At the molecular level, catecholamines downregulate the expression of B7–1 and major histocompatibility complex class I (MHC-I) molecules, while upregulating the expression of PD-1 and its ligand PD-L1, collectively suppressing the anti-tumor immune response within the TME (196). ACh represents another distinct mode of regulation. T cells themselves can produce ACh, which acts in an autocrine and paracrine manner on muscarinic acetylcholine receptors (mAChRs) and nicotinic acetylcholine receptors (nAChRs) on the surface of T cells, dendritic cells, and macrophages. This regulates cytokine production and immune function, thereby indirectly influencing cancer cell behavior. Mechanistically, ACh disrupts the recruitment of CD8^+^ T cells by inhibiting CCL5, thereby directly suppressing IFN-γ production by CD8^+^ T cells and promoting the differentiation of Th2 cells (201). Other neurotransmitters, such as 5-HT and CGRP, are also involved in remodeling the tumor immune microenvironment. 5-HT influences anti-tumor immunity by regulating dendritic cell function and T cell activation (196). CGRP, on the other hand, inhibits the activation of macrophages and T cells and promotes the production of TGF-β. In this way, CGRP prevents inflammation by inducing T cell apoptosis and macrophage-derived TGF-β, thereby fostering the formation of an immunosuppressive microenvironment (202). In summary, neurotransmitters play a central regulatory role in the remodeling of the tumor immune microenvironment through diverse receptor mechanisms and cell type-specific effects. A deep understanding of the molecular basis of neuro-immune axis will not only help reveal novel mechanisms of tumor immune evasion but also provide a theoretical foundation for the development of combination immunotherapies targeting neurotransmitter receptors.

We have summarized the enrichment of neurotransmitters in the TME [Sections 1.1, 1.2] and the essential role of DOCK2 and DOCK8 in immunological synapse formation [Sections 2.1, 2.3]. However, a direct mechanistic link connecting these observations remains to be established. In particular, while alpha2-AR are expressed on immune cells and can modulate cAMP/PKA signaling, there is currently no direct evidence that this pathway connects to DOCK family function or Rac/Cdc42 activation in the context of immunological synapse formation. We therefore do not include alpha2-AR signaling in our core mechanistic model, and we explicitly caution against inferring such a connection without direct experimental support.

### 5.2 Dendritic cell and macrophage motility paralysis in the TME

Mutant DOCK8 loses its ability to bind PI (4,5) P2 *in vitro*, thereby failing to activate Cdc42 and preventing leukocyte migration in three-dimensional collagen gels. Correspondingly, DCs carrying DOCK8 mutations are unable to undergo normal interstitial migration *in vivo* (49). DCs continuously adjust their morphology to adapt to the specific structure of the extracellular matrix, following the path of least resistance. However, DOCK8-deficient (DOCK8^-/-^) DCs fail to accumulate in the lymph node parenchyma to initiate T cell responses. Although DOCK8^-/-^ DCs migrate normally on two-dimensional surfaces, both their crawling within three-dimensional fibrillar networks and their migration through the floor of the subcapsular sinus require DOCK8 (48). When DCs encounter dense environments such as high-density collagen, they initiate the formation of a central F-actin pool. This central actin pool generates vertical propulsive forces that deform the matrix, creating a path for the cell body. DOCK8^-/-^ DCs completely lose the ability to form this central actin pool. Studies have confirmed that, in addition to reduced migration speed in collagen matrices, DOCK8^-/-^ DCs frequently undergo cell fragmentation due to the loss of the central actin pool that normally assists them in squeezing through narrow constrictions (203). Collectively, this evidence substantiates that DCs require DOCK8 to squeeze through and navigate dense tumor collagen. In summary, DOCK8, by mediating the formation of the central actin pool, endows DCs with the capacity to squeeze through dense tumor collagen networks. This molecular mechanism is crucial for DC infiltration into the TME and for initiating adaptive immune responses in lymph nodes, a conclusion supported by multiple lines of evidence.

Given the critical role of DOCK8 in enabling DCs to traverse dense collagen networks, it is tempting to speculate that neural signals might influence DC migration through DOCK8. Several studies have demonstrated that neurotransmitters can modulate DC function. For instance, γ-aminobutyric acid (GABA) has been reported to inhibit DC recruitment and T cell infiltration into tumors, partly through the GABA_B receptor and β-catenin signaling (204, 205). However, these studies do not establish a direct mechanistic link between GABA receptor signaling and DOCK8 phosphorylation, localization, or GEF activity in DCs.

A more specific connection between neural signaling and DOCK8 phosphorylation is not yet supported by direct experimental evidence. To date, no studies have directly demonstrated that neurotransmitters or neuropeptides modulate DOCK8 phosphorylation, Cdc42 activation, or F-actin architecture in DCs. Thus, while it remains an intriguing possibility that neural signals may regulate DC migration through DOCK8, this hypothesis requires direct experimental validation, particularly regarding DOCK8 phosphorylation status, membrane localization, and GEF activity in response to defined neural ligands. We therefore present this framework as a speculative hypothesis to guide future investigation, rather than as an established mechanism.

In the TME, macrophages are among the most abundant infiltrating immune cells and play a significant role in tumor initiation, progression, and metastasis. Macrophages can be classified into classically activated M1-type macrophages and alternatively activated M2-type macrophages (206). The functional polarization of TAMs is critical in determining their pro-tumor or anti-tumor phenotype. Pro-inflammatory M1-type macrophages possess the potential to phagocytose tumor cells; whereas anti-inflammatory M2-type macrophages, which predominate in tumors, promote tumor progression and mediate immune evasion (207). The processes of macrophage phagocytosis, migration, and polarization are highly dependent on the dynamic reorganization of the cytoskeleton, which is precisely regulated by Rho family GTPases (Rac/Cdc42). As key activators of Rho GTPases, aberrant signaling of DOCK family proteins can directly lead to macrophage functional reprogramming by disrupting actin dynamics. It is hypothesized that abnormalities in DOCK signaling not only impair the phagocytic capacity of macrophages but may also promote their polarization toward a pro-angiogenic M2 phenotype, thereby accelerating tumor progression.

**Figure 4:**
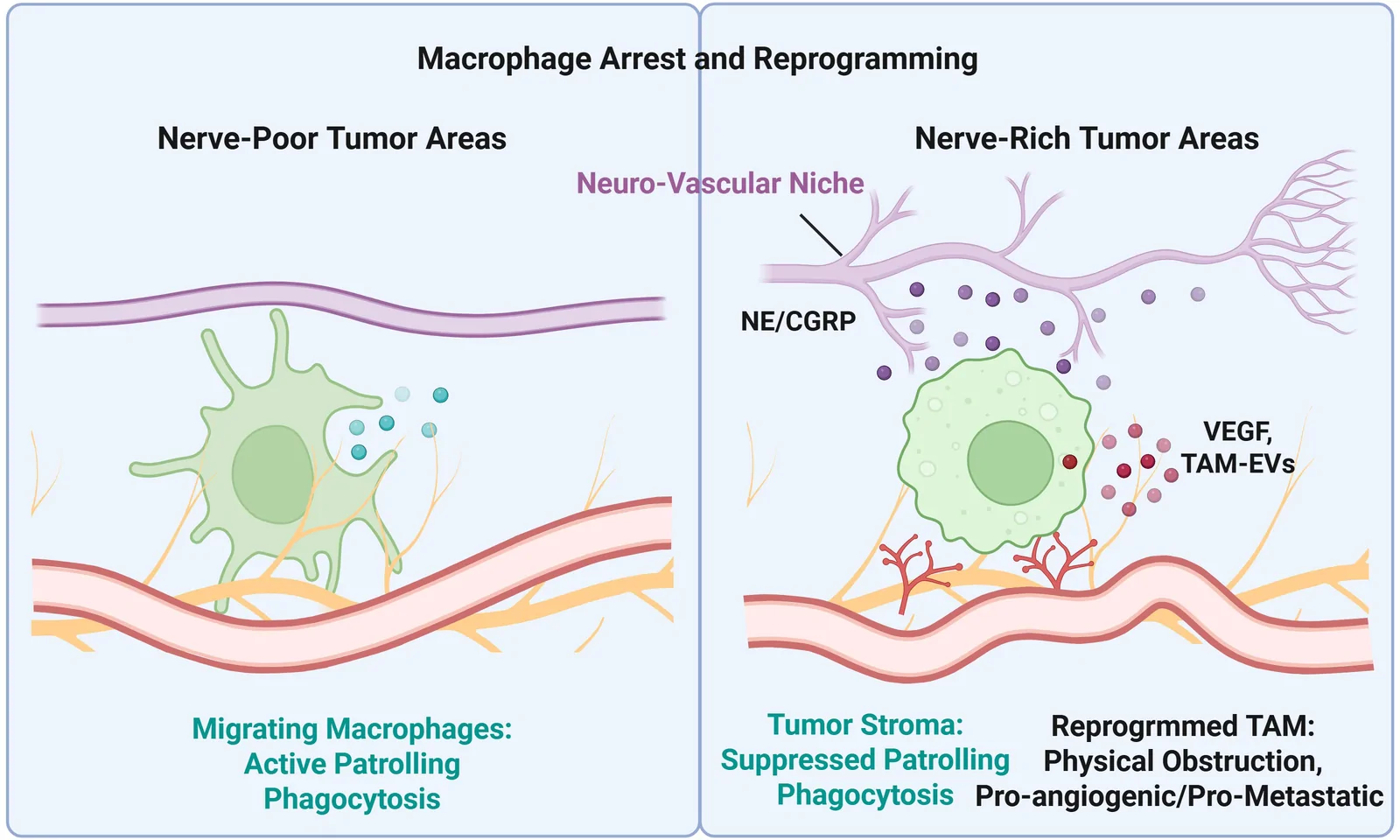
Neural signals induce macrophage reprogramming within the dense tumor stroma. Left panel: Macrophages in a poor innervation TME display robust migration, stromal patrolling, and tumor antigen phagocytosis. Right panel: In neuro-vascular niches, high neural signals paralyze macrophage motility. These arrested TAMs physically tether to nerves or vessels and undergo functional reprogramming, secreting pro-tumorigenic factors like VEGF and TAM-EVs to support angiogenesis and metastasis, rather than performing patrolling and phagocytosis.

Different members of the DOCK family often exert distinct functions in macrophages. This functional diversity suggests that aberrations in DOCK signaling may disrupt cytoskeletal dynamics, thereby driving the transition of macrophages from a migratory/phagocytic M1 phenotype to a pro-angiogenic, tissue-resident M2 phenotype. This transition is particularly critical within the TME and is exemplified by the distinct mechanisms through which DOCK7 and DOCK2 mediate macrophage functional reprogramming. On the one hand, DOCK7 acts as a key functional executor in TAMs. In colorectal cancer (CRC), TAMs directly promote tumor metastasis by secreting DOCK7-rich extracellular vesicles (TAM-EVs). The underlying mechanism involves DOCK7 protein encapsulated in TAM-EVs, which, upon entering tumor cells, activates Rac1. This subsequently upregulates ABCA1 expression via phosphorylation of AKT and FOXO1, thereby remodeling cholesterol metabolism in CRC cells and driving their metastatic behavior (208). This not only implicates DOCK7 signaling as central to TAM-mediated shaping of the pro-tumor microenvironment but also suggests DOCK7 as a novel potential therapeutic target for controlling CRC metastasis. On the other hand, in contrast to the pro-tumor role of DOCK7, DOCK2 is primarily involved in maintaining immune surveillance. In classical M1-type pro-inflammatory responses, DOCK2-mediated activation of the Rac-NF-κB pathway upon lipopolysaccharide (LPS) stimulation is crucial; its deficiency leads to impaired macrophage immune surveillance function (209). Therefore, it can be inferred that within TAMs, an imbalance in DOCK family signaling, particularly the loss of DOCK2 and aberrant high expression of DOCK7, disrupts Rac GTPase-mediated actin dynamics. This results in dual consequences: macrophages lose their phagocytic capacity and directional migration ability while being prompted to reside in the TME and acquire pro-angiogenic functions (**Figure 4**). This mechanistic chain provides a novel theoretical basis for targeting the “DOCK-cytoskeleton axis” to reverse the pro-tumor phenotype of TAMs.

## 6 Conclusion and future perspectives

While our model emphasizes that chronic neural signals promote an immunologically “cold” TME, it is important to acknowledge that the relationship between NE and CD8^+^ T cell function is context-dependent. Acutely, NE can act as a pro-inflammatory stimulus, preferentially inducing inflammatory cytokine production in memory CD8^+^ T cells via the β2-adrenergic receptor (ADRB2) (210). However, the tumor microenvironment is characterized by sustained, rather than transient, neurotransmitter exposure. Under such chronic conditions, NE drives CD8^+^ T cells toward exhaustion, marked by PD-1 upregulation and impaired effector function (211). Notably, recent evidence suggests that exhausted CD8^+^ T cells themselves upregulate ADRB1 and physically cluster around sympathetic nerves, creating a positive feedback loop that perpetuates their dysfunctional state. Thus, in the highly innervated TME, the net consequence of chronic neural signaling is the promotion of T cell exhaustion and the establishment of a “cold” TME, outweighing any transient pro-inflammatory effects.

In this review, we synthesize current evidence supporting a working framework in which neuroimmune signals may converge on DOCK-family cytoskeletal programs to modulate immune cell function in solid tumors. We focus on the established roles of DOCK2 and DOCK8 in immune cell trafficking, immune synapse formation, and Rac/Cdc42-mediated actin remodeling, and we explore the emerging evidence that neural ligands within the TME could potentially influence these cytoskeletal programs through receptor-mediated signaling.

Immune cells widely express neurotransmitter receptors that can modulate their function. β-AR signaling has been reported to promote M2-like macrophage polarization, and CGRP may impair DC antigen presentation and promote T cell exhaustion. In T cells, β2-AR activation increases cAMP and PKA activity. It has been hypothesized that PKA might phosphorylate DOCK-family GEFs, but direct evidence for this in immune cells is lacking. Moreover, structural studies indicate that DOCK2-ELMO1 phosphorylation can favor an active GEF conformation, which is difficult to reconcile with a generalized inhibitory model. The functional consequences of PKA-mediated phosphorylation on DOCK proteins therefore appear to be isoform-specific and context-dependent. Whether such phosphorylation events occur in immune cells within the TME, and whether they lead to activation or inhibition, remains an open question requiring direct experimental investigation. Isoform-specific evidence for DOCK phosphorylation is summarized in **Table 2**.

**Table 2:** Isoform-specific evidence for DOCK phosphorylation.

DOCK isoform	Cell type/context	Kinase	Phosphorylation site	Effect on GEF activity	Evidence level	Key references
DOCK1/Dock180	Glioma cells	PKA	Ser1250	Activating (promotes Rac1 activation)	Direct (in tumor cells)	(178)
		SRC family kinases	Tyr722			(188)
DOCK2	*In vitro* (structural)	Not identified	Not specified	Activating (destabilizes auto-inhibited state, favors active GEF conformation)	Structural (inferred)	(47)
DOCK5	—	Not identified	Ser365, Ser1656, Ser1756 (database records)	Not determined	Absent (functional validation lacking)	—
DOCK8	T cells	PKCα (not PKA)	Ser2077, Ser2082, Ser2087	Activating (promotes Cdc42 activation and T cell migration)	Direct (but not PKA)	(189)

“Direct” indicates experimental evidence from the cited study; “Structural” indicates evidence from crystallographic/cryo-EM studies; “Inferred” indicates a plausible but unconfirmed mechanism; “Absent” indicates no direct evidence currently available; “—” indicates no available data or no functionally validated literature.

It is important to note, however, that the regulation of immune cells by sympathetic signals is likely context-dependent and may involve multiple adrenergic receptor subtypes. While beta2-AR and alpha2-AR signaling have opposing effects on the cAMP-PKA axis, the convergence of these pathways on DOCK inhibition, as previously hypothesized, is not supported by direct experimental evidence. We have therefore removed this speculative model and focus instead on the beta2-AR-cAMP-PKA pathway, where the available evidence provides a clearer, albeit still incomplete, basis for further investigation. Several critical links in this proposed axis, particularly the direct connection between specific neural receptors and PKA-mediated DOCK phosphorylation in the TME, remain to be experimentally confirmed. Direct biochemical or structural evidence is lacking for most of the proposed steps.

The pathological significance of this proposed neuro-immune-cytoskeleton axis may be particularly relevant in highly innervated tumors, such as neuroblastoma, where abundant nerve infiltration could potentially enhance immune evasion through both local neurotransmitter release and systemic neural modulation of distal immune organs. However, direct evidence for this model is currently lacking. To guide future investigation, we outline several key experiments needed to test the proposed framework: stimulation of tumor-infiltrating lymphocytes, dendritic cells, or tumor-associated macrophages with defined neural ligands to assess DOCK activity; phospho-profiling of DOCK isoforms; membrane recruitment and Rac/Cdc42 activity assays; F-actin imaging and immune-synapse assays; 3D matrix migration assays; and rescue experiments with isoform-specific DOCK mutants. Until such evidence becomes available, the model presented here should be viewed as a testable framework to guide future research.

**Figure 5:**
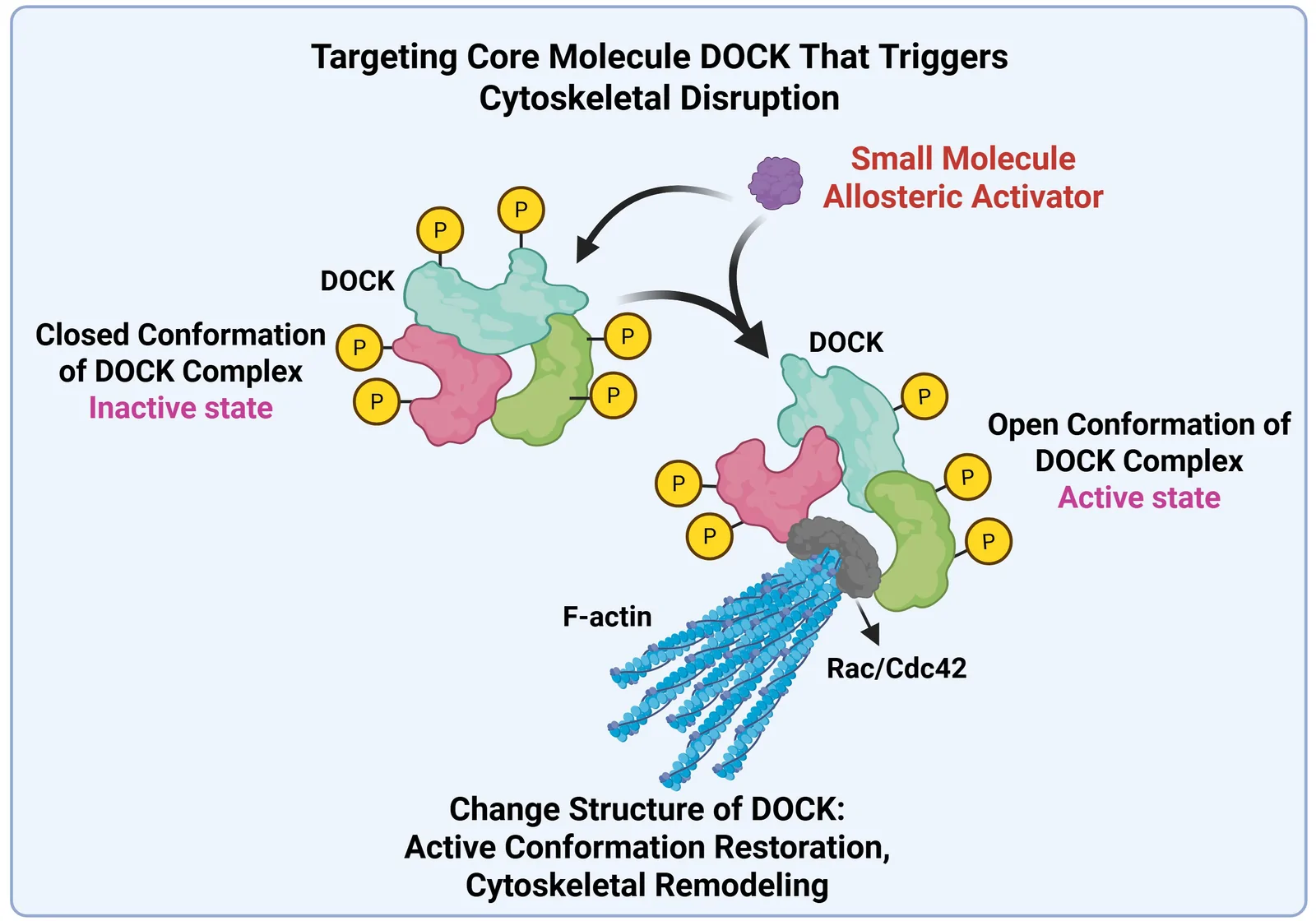
Speculative future directions: targeting DOCK-family GEFs as a therapeutic strategy. Targeting DOCK, a key molecule positioned in the midstream of the signaling axis, we propose the hypothetical concept of developing an allosteric modulator that could modulate DOCK activity. This proposed approach remains speculative and requires extensive experimental validation.

Based on this proposed framework, each node in this cascade could be viewed as a potential target for therapeutic intervention (**Table 3**). However, it is important to distinguish between approaches with existing evidence and those that remain speculative. β-blocker repurposing and denervation strategies have shown promise in preclinical models and, in some cases, in human studies, and these are discussed as plausible near-term translational opportunities. By contrast, concepts such as DOCK allosteric modulators and small-molecule agents targeting downstream effectors such as WASp and PAK are currently hypothetical. These ideas may be interesting as future directions, but they require extensive basic mechanistic validation, including identification of the relevant DOCK isoform, phosphorylation sites, direction of effect, and safety considerations, before any translational consideration can be justified (**Figure 5**).

**Table 3:** Established and prospective clinical interventions targeting the “neuro-immune-cytoskeleton” signaling axis.

Precision targeting approach at which level of the signaling axis does the drug target?	Therapeutic agents/interventional preparations drug class/name	Underlying mechanism focus on strategies for cytoskeletal dynamics restoration (established and hypothetical approaches)	Target solid tumors high innervated tumor models	Clinical/preclinical status select examples/NCT
Upstream blockade of the interaction between neurotransmitters and β-ARs	Non-specific β-blockers (propranolol, atenolol)	(1) Competitively inhibit neurotransmitters (e.g., NE/EPI) binding to β-ARs on immune cells; (2) Reduce intracellular cAMP/PKA signaling activity; (3) Modulate downstream signaling pathways; direct evidence linking β-blockers to DOCK/ELMO phosphorylation is currently lacking; (4) Have been shown to enhance immune cell motility in some contexts, but DOCK-specific restoration of actin polymerization remains unconfirmed.	(1) Melanoma (212) (2) Breast Cancer (213) (3) Pancreatic Ductal Adenocarcinoma (PDAC) (28)	Phase Ib/II trial of propranolol plus pembrolizumab in advanced melanoma (NCT03384836; currently suspended) (214).
Upstream blockade the binding between neuropeptides (peptidergic) and their receptors	CGRP receptor antagonists (mAbs: Fremanezumab; Gepants: Ubrogepant)	(1) Block CGRP binding to its receptors on DCs/T cells; (2) Attenuate CGRP-mediated cAMP/PKA signaling; (3) May relieve CGRP-induced suppression of DC migration and antigen presentation, but direct evidence for DOCK-specific effects is currently lacking.	(1) Melanoma (31) (2) Breast Cancer (108)	FDA-approved for migraine; currently in preclinical studies for oncology (no clinical data in cancer patients reported to date).
Physical/Surgical Denervation	(1) Nerve resection via surgery (2) Local neurotoxin administration (botulinum toxin/Botox; resini feratoxin/RTX)	(1) Disrupts neurotransmitter release in the TME; (2) Attenuates nerve-mediated immunosuppression (e.g., reduces T cell exhaustion, promotes M1 polarization); direct evidence for cytoskeletal effects is currently lacking; (3) Has been associated with increased T cell infiltration in certain models; whether this reflects restored infiltration capacity remains to be directly demonstrated.	(1) Prostate Cancer (215) (2) Gastric Cancer (216, 217).	Human prostate cancer was BoNT-A chemically denervated (NCT01520441) (218). BoNT-A injection in gastric cancer treatment. (NCT01822210)
Cytoskeletal Regulation (Prospective concept: Hypothetical/Discovery stage)	(1) DOCK5 allosteric inhibitor (C21) (219) (2) DOCK3 allosteric activators (220)	(1) Non-competitive inhibitor that remodels DOCK5-Rac1 complex into unproductive conformation; (2) Enhance DOCK3-Elmo1 interaction, promote Rac1 activation and neurite outgrowth.	N/A	Preclinical (*in vitro*/neuroprotection models only); no evidence in tumor immunology.
Cell Therapy	Armored CAR-T/NK cells (e.g., DOCK8/CDC42-overexpressing CAR-T)	Overexpression of DOCK8 or CDC42 has been proposed to enhance CAR-T cell function in patent literature [US20200172865A1/CN110819596A/CN117165532A]; the specific concept of phospho-resistant DOCK mutants resisting neural signals is hypothetical.	Patent-proposed strategy for various solid tumors; currently at preclinical/patent stage with no published *in vivo* or clinical validation in specific tumor types.	Preclinical/patent stage (*in vitro* evidence for DOCK8/CDC42 overexpression in CAR-T cells from patent literature; no *in vivo* or clinical validation; phospho-resistant DOCK mutants remain hypothetical).

**Figure 6:**
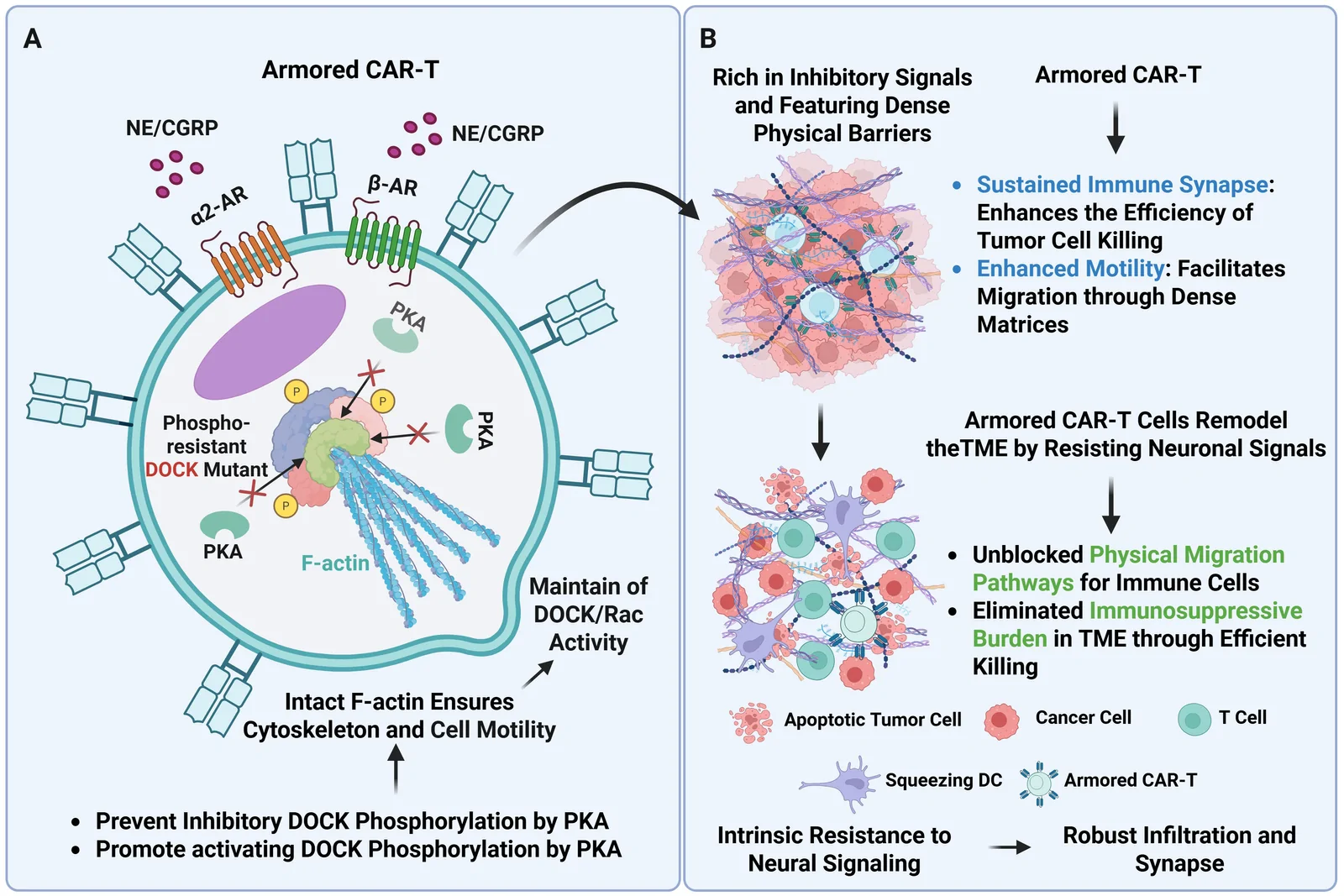
Speculative future directions: a purely hypothetical concept for DOCK-engineered armored CAR-T cells. **(A)** Hypothetical design of armored CAR-T cells engineered with phospho-resistant DOCK mutants, proposed to sustain F-actin polymerization and cytoskeletal integrity upon neurogenic signaling. **(B)** Proposed functional advantages in the TME: enhanced matrix penetration and preserved immune synapse formation. This concept remains entirely hypothetical and has not been experimentally validated; it is discussed only as a speculative future direction.

More speculative strategies, such as DOCK-engineered CAR-T or CAR-NK cells designed to resist phosphorylation-mediated inactivation, may also be conceptually interesting as future directions, but they face the same requirement for extensive mechanistic validation before any translational consideration can be justified (**Figure 6**).

In summary, this review synthesizes current evidence supporting a working hypothesis that neuroimmune signals may converge on DOCK-family cytoskeletal programs to modulate immune cell function in solid tumors. By mapping the established functions of DOCK2 and DOCK8 in immune cell trafficking, synapse formation, and actin remodeling, and by reviewing emerging evidence for tumor innervation and neurotransmitter receptor signaling, we provide a conceptual framework that may help guide future investigation. If validated, this framework could offer new therapeutic opportunities for overcoming immune resistance. At present, however, the model remains a testable hypothesis, and we have explicitly acknowledged the evidence gaps that must be addressed before it can be considered established.

## Glossary

TME: tumor microenvironment
DOCK: dedicator of cytokinesis
GEFs: guanine nucleotide exchange factors
DCs: dendritic cells
ISs: immune synapses
MSCs: mesenchymal stem cells
ECM: extracellular matrix
CNS: central nervous system
PNS: peripheral nervous system
NTFs: Neurotrophic factors
ICB: immune checkpoint blockade
NE: Norepinephrine
Tregs: regulatory T cells
MDSCs: myeloid derived suppressor cells
NK: natural killer
PD-L1: programmed death-ligand 1
PD-1: programmed cell death protein 1
IHC: immunohistochemistry
EMT: epithelial-mesenchymal transition
EPI: epinephrine
AR: adrenergic receptor
TAMs: tumor-associated macrophages
CGRP: Calcitonin gene-related peptide
RAMP1: receptor activity-modifying protein 1
LCs: langerhans cells
SP: Substance P
NK-1R: neurokinin-1 receptor
SNS: sympathetic nervous system
cDC1s: Conventional type 1 dendritic cells
DHR: DOCK homologous region
SLOs: secondary lymphoid organs
PLNs: peripheral lymph nodes
TCR: T cell receptor
BCR: B cell receptor
GPCR: G protein coupled receptor
NETs: neutrophil extracellular traps
STAD: stomach adenocarcinoma
TCM: central memory T cell
AR-HIES: autosomal recessive high immunoglobulin E syndrome
LFA-1: lymphocyte function associated antigen 1
WASp: Wiskott Aldrich syndrome protein
WIP: Wiskott Aldrich syndrome protein-interacting protein
MTOC: microtubule-organizing center
Tfh: T follicular helper
GCs: germinal centers
HPV: human papillomavirus
cAMP: cyclic adenosine monophosphate
PKA: protein kinase A
AKAPs: A-kinase anchoring proteins
ELMO1: engulfment and cell motility 1
APCs: antigen-presenting cells
cSMAC: central supramolecular activation cluster
pSMAC: peripheral supramolecular activation cluster
dSMAC: distal supramolecular activation cluster
NDDs: neurodegenerative diseases
ACh: acetylcholine
MHC-I: major histocompatibility complex class I
mAChRs: muscarinic acetylcholine receptors
nAChRs: nicotinic acetylcholine receptors
GABA: γ-aminobutyric acid
CRC: colorectal cancer
EVs: extracellular vesicles
LPS: lipopolysaccharide
